# The Rise of Intelligent Plastic Surgery: A 10-Year Bibliometric Journey Through AI Applications, Challenges, and Transformative Potential

**DOI:** 10.1007/s00266-025-05068-4

**Published:** 2025-07-14

**Authors:** Yi Liu, Kexin Deng, Chengwu Zhang, Zhigen Yuan, Jianda Zhou, Can Liu

**Affiliations:** 1 Department of Plastic Surgery, Central South University, The Third Xiangya Hospital, Changsha, 410013 Hunan China; 2 Department of Orthopaedics, Brain Hospital of Hunan Province (The Second People’s Hospital of Hunan Province), 427#, Furong Road, Changsha, 410007 Hunan China

**Keywords:** Bibliometrics, CiteSpace, Plastic surgery, Artificial intelligence (AI), Visualization, Surgical decision-making

## Abstract

**Background:**

Driven by advancements in deep learning, surgical robots, and predictive modeling technologies, the integration of artificial intelligence (AI) and plastic surgery has expanded rapidly. Although AI shows the potential to enhance precision and efficiency, its clinical integration faces challenges, including ethical concerns and interdisciplinary complexity, which require a systematic analysis of research trends.

**Methods:**

The CiteSpace and VOSviewer software were used to conduct a quantitative analysis of 235 documents in the core collection of Web of Science from 2016 to 2024. Co-citation networks, keyword co-occurrence, burst detection, and cluster analysis were employed to map the research trajectories. The inclusion criteria gave priority to studies that explicitly incorporated artificial intelligence into surgical designs or outcomes. The contributions of countries, institutions, and authors were evaluated through centrality indicators.

**Result:**

Publications related to artificial intelligence have grown exponentially, with the USA, Germany, and Canada leading research output. Harvard and Stanford Universities dominate in terms of institutional contributions, but cross-institutional collaboration remains limited. The keyword cluster highlights the innovations of artificial intelligence in breast reconstruction, facial analysis, and automated grading systems. Burst terms such as “deep learning,” “risk assessment,” and “attractiveness” underscore AI’s role in optimizing surgical outcomes, but they also expose biases against Western-centric beauty standards. Ethical concerns, dataset diversity gaps, and overreliance on AI-driven decisions have become key obstacles.

**Conclusion:**

The integration of artificial intelligence in plastic surgery goes beyond the utility based on tools and into data-informed surgical engineering. The persistent gap in collaboration and dataset diversity highlights the need for global, interdisciplinary efforts to address technical and ethical challenges while advancing AI’s clinical utility. Future research must prioritize transparency, inclusivity, and collaborative innovation to realize AI’s transformative potential while mitigating risks.

**Level of Evidence IV:**

This journal requires that authors assign a level of evidence to each article. For a full description of these Evidence-Based Medicine ratings, please refer to the Table of Contents or the online Instructions to Authors www.springer.com/00266.

**Supplementary Information:**

The online version contains supplementary material available at 10.1007/s00266-025-05068-4.

## Introduction

Given the substantial market demand, which spans from “correcting physical contours or physiological defects” to “enhancing quality of life,” the role of plastic surgery within the healthcare framework has become increasingly pivotal [1–3]. Driven by advancements in multidisciplinary technologies—such as regenerative medicine, tissue engineering [4, 5], and the rise of minimally invasive and non-invasive techniques [6]—the traditional paradigm of plastic surgery is undergoing a transformative evolution [7, 8].

AI aims to simulate human cognitive functions through data and algorithms [9] and has evolved from its early exploratory phase with limited computational power to the current era of deep learning and large-scale models [10, 11], which are now adept at processing diverse medical datasets [12]. AI applications have progressed from simple algorithm-based care to sophisticated models for disease diagnosis and personalized medicine [13]. The integration of AI into surgical practice has garnered significant academic attention. Breakthroughs in AI applications for medical imaging analysis [14], surgical robotics [15], and prognostic prediction [16] have surpassed its conventional role as a tool [17]. Currently, although the integration of AI in plastic surgery is a relatively recent development, its advanced capabilities in spatial imaging, data processing, and learning have driven rapid progress and application within the field. This is particularly evident in areas such as 3D imaging, surgical planning, risk assessment, real-time intraoperative assistance, and postoperative outcome prediction.

For instance, procedures such as facial rejuvenation and breast reconstruction, AI demonstrates the capability to identify anatomical landmarks and facilitates three-dimensional visualization of predicted surgical outcomes [18, 19]. Using VoNavix software combined with CTA to construct 3D vascular images, through delivering precise visual detail, supports intraoperative decision-making in free abdominal flap breast reconstruction [20]. And in the field of lymphatic microsurgery, surgical robotic assistance enhances accessibility to deep anatomical regions, improving procedural precision and reducing operative duration [21, 22]. Based on deep learning technology, a University of California research team developed a deep convolutional neural network termed RhinoNet, achieving 85% accuracy in predicting rhinoplasty outcomes [23]. These capabilities perfectly align with the requirements of body and facial contour remodeling, showcasing substantial potential for clinical integration in plastic surgery while probably reshaping the entire diagnostic and therapeutic paradigm [24].

The literature pertaining to AI applications in aesthetic and reconstructive surgery is both expansive and heterogeneous. To delineate the clinical impact of technological advancements, a macro-level bibliometric analysis is warranted to systematically map the structural composition and research priorities across this domain. Bibliometric methodologies enable quantitative field assessment [25], evaluation of academic contributions from nations, institutions, journals, and investigators [26], and identification of evolving research fronts and emergent trends [27]. To date, however, no comprehensive analysis has mapped AI's evolution within plastic surgery using this approach. This study employs bibliometric and visual analytics to synthesize the developmental trajectory, critical knowledge gaps, current landscape, and future directions of AI integration in plastic surgery over the past decade.

## Methods

### Research Methodology

This study employed bibliometric methods and utilized CiteSpace software to analyze research literature on the application of artificial intelligence in plastic and aesthetic surgery [28, 29]. The relationships among the literatures were explored through co-citation analysis, and the evolution of research hotspots was tracked by using keyword co-occurrence and burst detection techniques [30]. Clustering analysis facilitated thematic categorization. By integrating co-citation networks, co-occurrence patterns, burst detection, and cluster analysis, we mapped the knowledge structure of this field. Key nodes linking disparate research themes were identified using mediation centrality (threshold >0.1). Through a synthesis of quantitative and qualitative approaches, this study systematically reviewed the current state, developmental trajectory, and future prospects of AI in plastic surgery [31].

### Data Resource

In this paper, Web of Science Core Collection (WoSCC) was selected and the index was Science Citation Index Expanded (SCIE). The retrieval formula in this paper was as follows TS=((brow* lift*) OR (ear* surg*) OR (eyelid* surg*) OR (facelift*) OR (facial* bone* cont*) OR (lip* augment*) OR (front* surg*) OR (rhinoplas*) OR (buttock* augmentat*) OR (buttock* lift*) OR (liposuct*) OR (thigh* lift*) OR (arm* lift*) OR (labiaplast*) OR (vaginal* rejuvenat*) OR (breast* augment*) OR (breast* implant* removal*) OR (breast* lift*) OR (breast* reduct*) OR (gynecomastia*) OR (Plastic* Surg*) OR (Reconst* Surg*) OR (Cosmetic* Surg*) OR (Mastop*) OR (Abdominop*) OR (Blepharop*) OR (Derm* Filler*) OR (Scar* Revis*) OR (Cleft* Lip* and Palat* Repair*) OR (Aesthetic* Surg*) OR (Body* Lift*) OR (Forehead* Lift*) OR (Keloid*) OR (Fat* Graft*) OR (Nasal* Reconstruct*) OR (Tummy* Tuck*) OR (Vaginop*) OR (Pectoral* Implant*) OR (Abdominal* Contour*) or (Aesthetic* Procedure*)) AND TS= (artificial* intelligen* OR open* ai OR intelligen* artificielle* OR chatgpt*). All search terms were determined according to the medical procedures included in the 2024 Survey Report on Aesthetic Plastic Surgery published by The International Society of Aesthetic Plastic Surgery (ISAPS) [32]. All electronic searches were performed on January 1st, 2025 in China. A total of 1605 articles were searched, 1068 original articles were selected and written in English. We subsequently conducted a triple-blind screening of the initially included literature based on the following inclusion and exclusion criteria. To ensure the accuracy and relevance of the literature, we used the intersection of the literature selected by the three reviewers to conduct subsequent analyses. The three reviewers are PhDs in Plastic and Aesthetic Surgery from three different provinces in China.

**Fig. 1:**
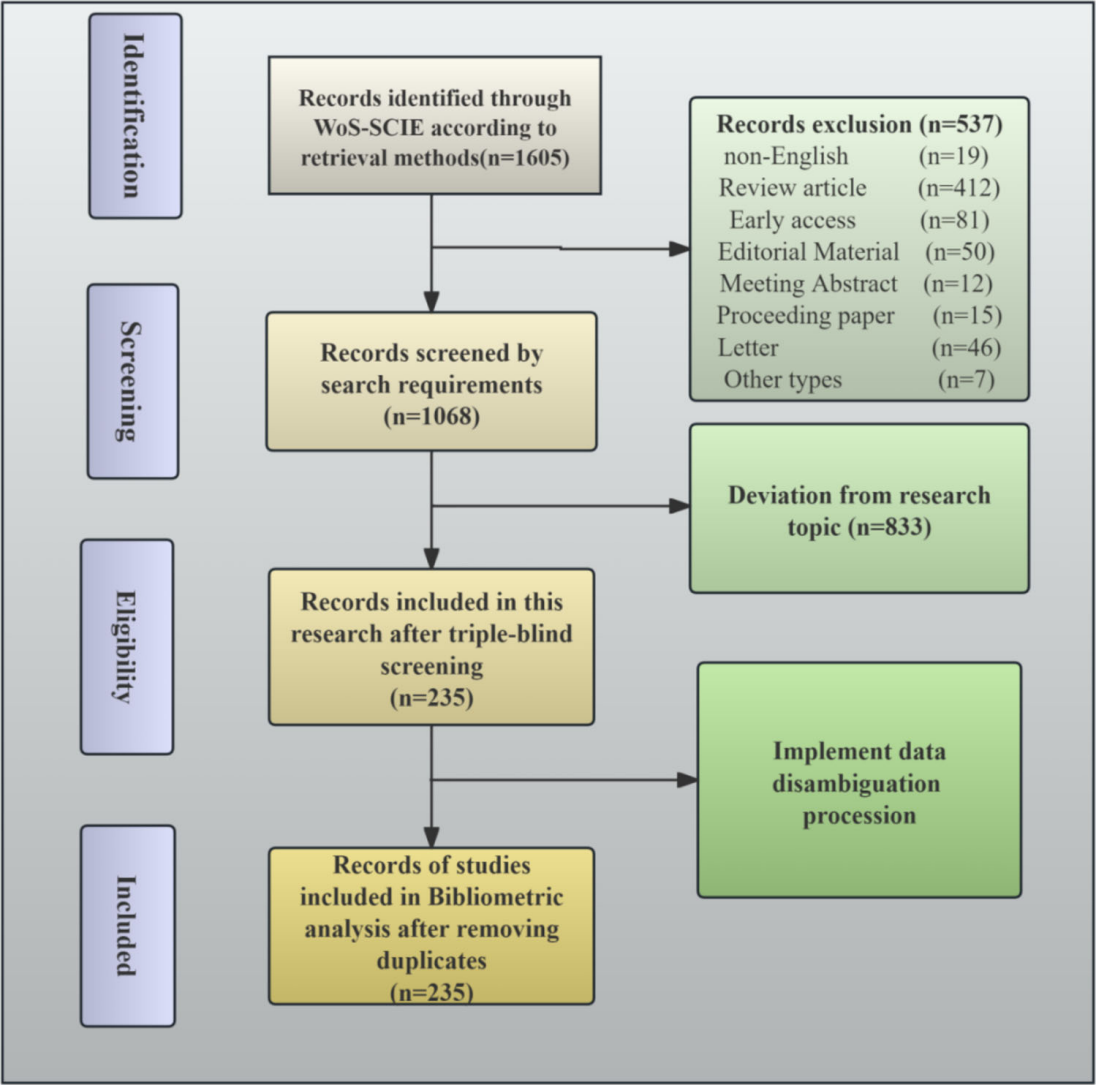
The search strategy used for the present bibliometric analysis by PRISMA

Finally, this paper performed a bibliometric analysis of the 235 articles after removing duplicated articles. The whole literature retrieval and screening process using PRISMA flowchart is shown in Fig. 1.

### Inclusion Criteria

1. Search is limited to English publications with complete reference and citation records.
2. Studies must focus on plastic and aesthetic surgery, including but not limited to medical imaging for plastic surgery, surgical procedures, disease diagnosis, complications, anatomy, and therapeutic outcome evaluation.
3. Studies must explicitly involve artificial intelligence (AI) or its core components, such as neural networks, deep learning, natural language processing (NLP), or large-scale database mining.

### Exclusion Criteria

1. Non-English publications, articles with incomplete reference or citation records.
2. Studies involving non-human subjects (e.g., dogs, cattle, cats, pigs).
3. Articles that merely mention AI incidentally (e.g., statements like "this abstract/body text was generated by AI and manually revised" or "future studies could explore AI applications...") without integrating AI into the research design, methodology, or objectives.
4. Articles containing keywords related to plastic and aesthetic surgery (e.g., "facial feminization surgery," "breast augmentation"), but addressing topics unrelated to the discipline (e.g., veterinary surgery, material science).

## Results

### National, Institutional, Author, and Journal Analysis

#### Analysis of Publication Trends

**Fig. 2:**
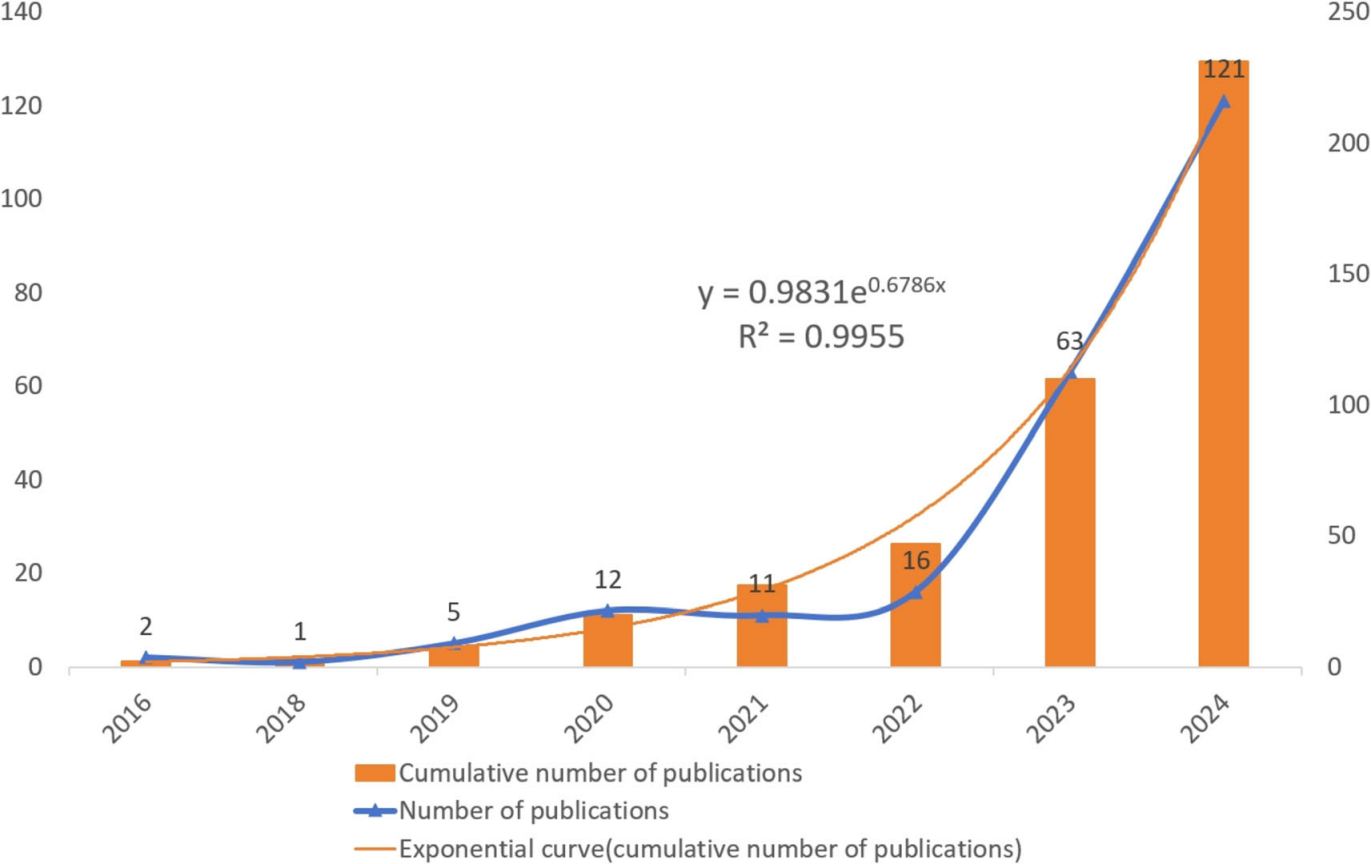
Time evolution of the total number of publications in the WOS database

The annual volume of publications in a specific field serves as a critical indicator of its research status and future trajectory. By plotting the publication count-time curve, we observed that the application of artificial intelligence in plastic surgery has grown exponentially over the past 3 years (Fig. 2). This trend suggests the sustained momentum in the field and is expected to further develop in the coming years.

### National Analysis

**Fig. 3:**
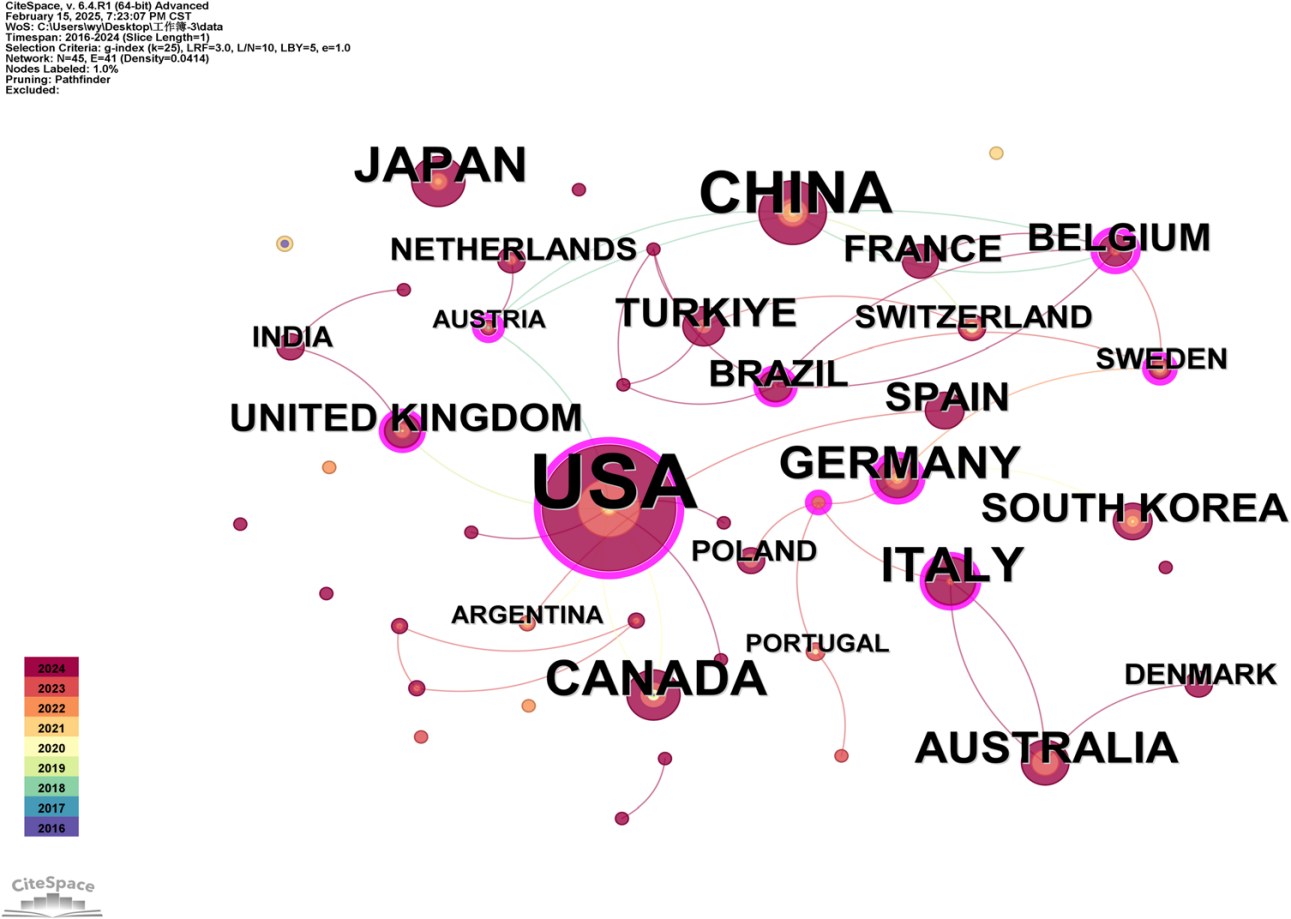
Analysis of publication and Cooperation network of countries. The “Time Slicing” was set to “2016–2024” with a “Years Per Slice” of “1.” In the visualization, the purple outer ring represents countries with an intermediary centrality greater than 0.1. Intermediary centrality is a measure of the importance of the research object in the collaborative network, indicating the strength of its bridging role in connecting multiple other countries. The size of the nodes corresponds to the volume of publications, with countries that have a higher number of publications being represented by larger nodes

Quantitative analysis of national contributions has identified the leading countries driving research in the intersection of AI and plastic surgery. All of these countries have played important roles in international academic exchanges and the publication of results. As illustrated in Fig. 3 and Table 1, the USA holds a dominant position, which reflects its strong academic interest and leadership in both AI and plastic surgery. The high centrality value (mediating role in academic networks) of the USA underscores its pivotal role in facilitating global scholarly exchanges. Germany (centrality = 0.37), as the largest economy in Europe, also plays a significant role in international collaboration. Canada was the first country to adopt artificial intelligence for plastic surgery. Overall, research output is predominantly concentrated in developed nations, including the US, Japan, and EU countries.

**Table 1:** The frequency and centrality of publication in countries/regions

Country	The first publication year	Frequency	Centrality
USA	2016	104	0.45
China	2018	33	0.08
Japan	2021	17	0
Canada	2016	16	0
Italy	2023	15	0.11
Germany	2020	13	0.37
Australia	2023	12	0.06
South Korea	2020	9	0
Spain	2023	9	0
Turkey	2023	9	0.01

### Institutional Analysis

**Fig. 4:**
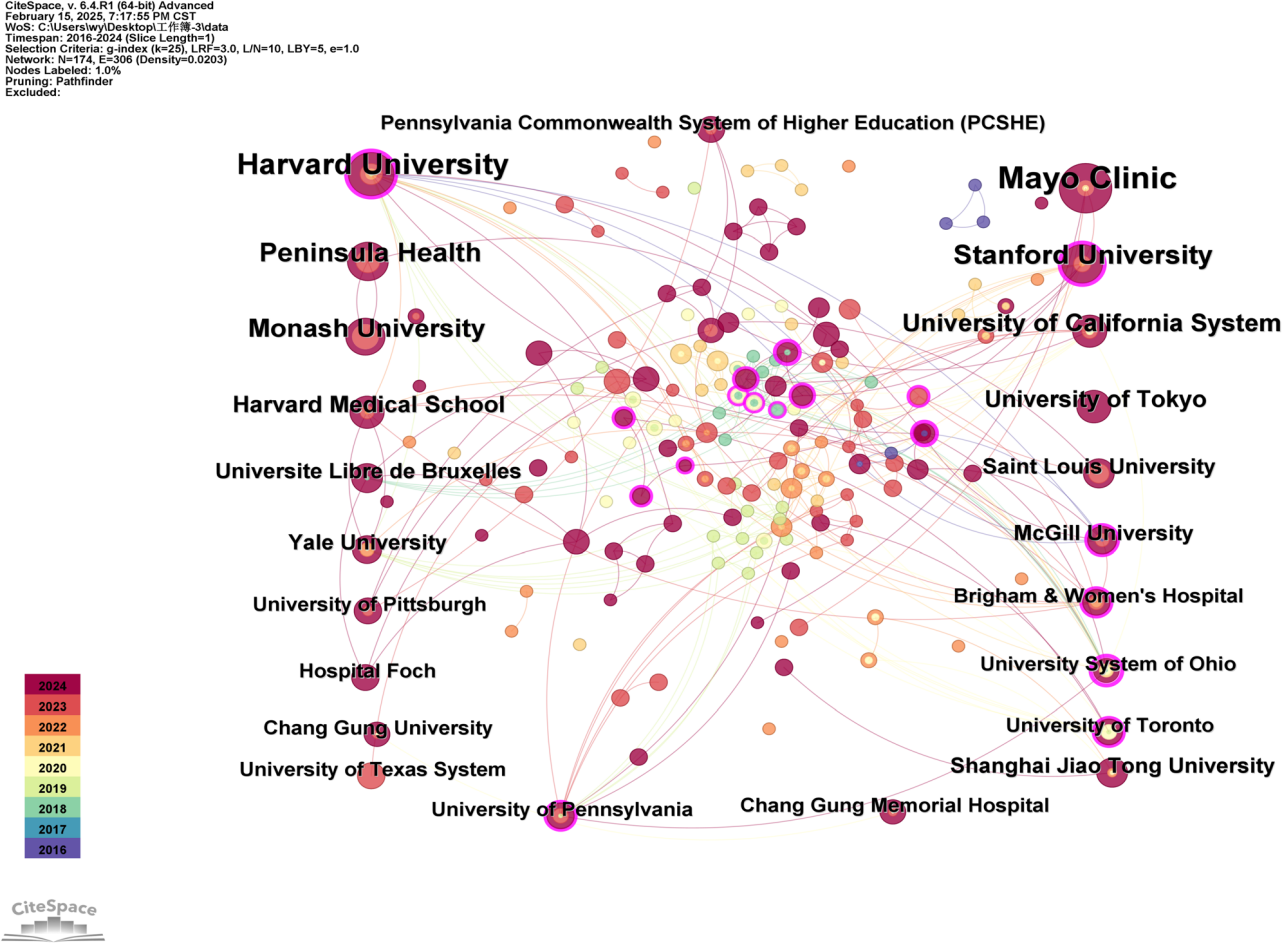
Institutional co-occurrence map. Featuring 174 network nodes, 306 connections, and a density of 0.0203. Other information is consistent with the analysis image of national cooperation

An institutional co-occurrence network was generated using CiteSpace (Fig. 4), highlighting the top 15 contributing institutions (Table 2). Two-thirds of these institutions are located in the USA. It was worth noting that Harvard University (centrality = 0.27) and Stanford University (centrality = 0.31) were the only institutions with a centrality value exceeding 0.25, underscoring the leading position of the USA in international academia. Among the remaining 13 institutions, only two had a centrality value exceeding 0.1, indicating limited cross-institutional collaboration and a tendency toward independent research. The overall low publication volume and pronounced regional clustering—consistent with national-level findings—partially hinder the field’s rapid development.

**Table 2:** The top 15 institutions of publication

Institution	First publication year	Frequency	Centrality
Mayo Clinic	2020	16	0.03
Harvard University	2016	14	0.27
Stanford University	2021	11	0.31
Peninsula Health	2023	10	0
Monash University	2023	9	0
University of California System	2020	8	0.09
University of Tokyo	2024	7	0
Harvard Medical School	2022	7	0.02
Shanghai Jiao Tong University	2020	6	0
McGill University	2016	6	0.13
Saint Louis University	2023	6	0.01
Yale University	2019	6	0.01
Universite Libre de Bruxelles	2018	6	0.07
University of Toronto	2019	5	0.11
University of Pennsylvania	2019	5	0.15

### Analysis of Authors

**Fig. 5:**
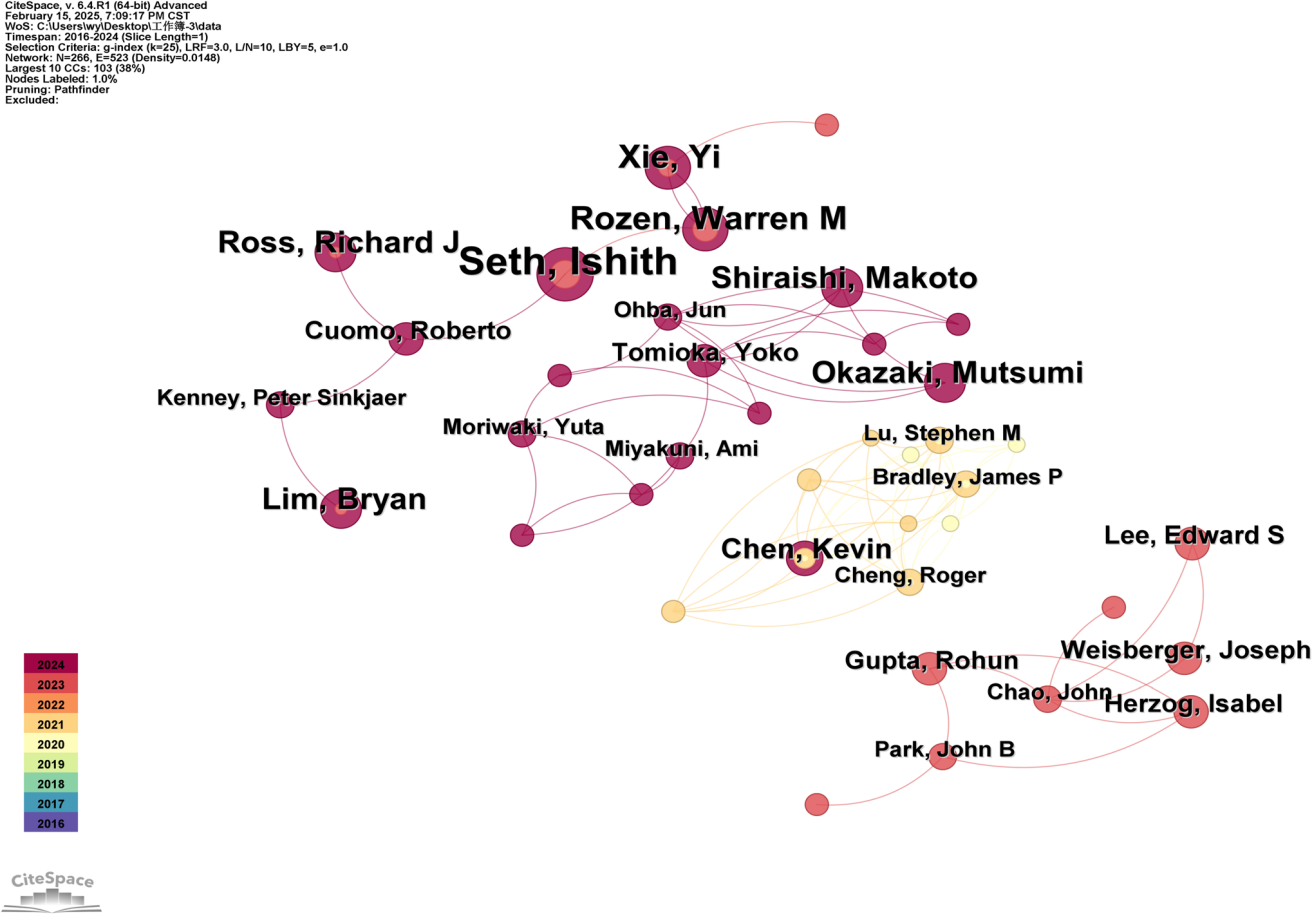
Analysis diagram of author collaboration network. Featuring 266 network nodes, 523 connections, and a density of 0.0148. Other information is consistent with the analysis image of national cooperation

Author contribution analysis (Fig. 5) identified the top 15 researchers in the field (Table 3). The median centrality value of no author is greater than 0.1, indicating that they tend to conduct independent research and lack in-depth cooperation. However, these authors could be recognized as pioneers in the field, reflecting its current developmental constraints.

**Table 3:** Top 15 authors in publication count

Authors	First publication years	Frequency
Seth, Ishith	2023	11
Rozen, Warren M	2023	7
Xie, Yi	2023	7
Ross, Richard J	2023	6
Okazaki, Mutsumi	2024	6
Shiraishi, Makoto	2024	6
Lim, Bryan	2023	6
Lechien, Jerome R	2024	5
Borna, Sahar	2024	5
Chen, Kevin	2020	5
Asaad, Malke	2023	5
Gomez-cabello, Cesar A	2024	5
Haider, Syed Ali	2024	5
Pressman, Sophia M	2024	5
Herzog, Isabel	2023	4

### Journal Co-Citation Analysis

**Fig. 6:**
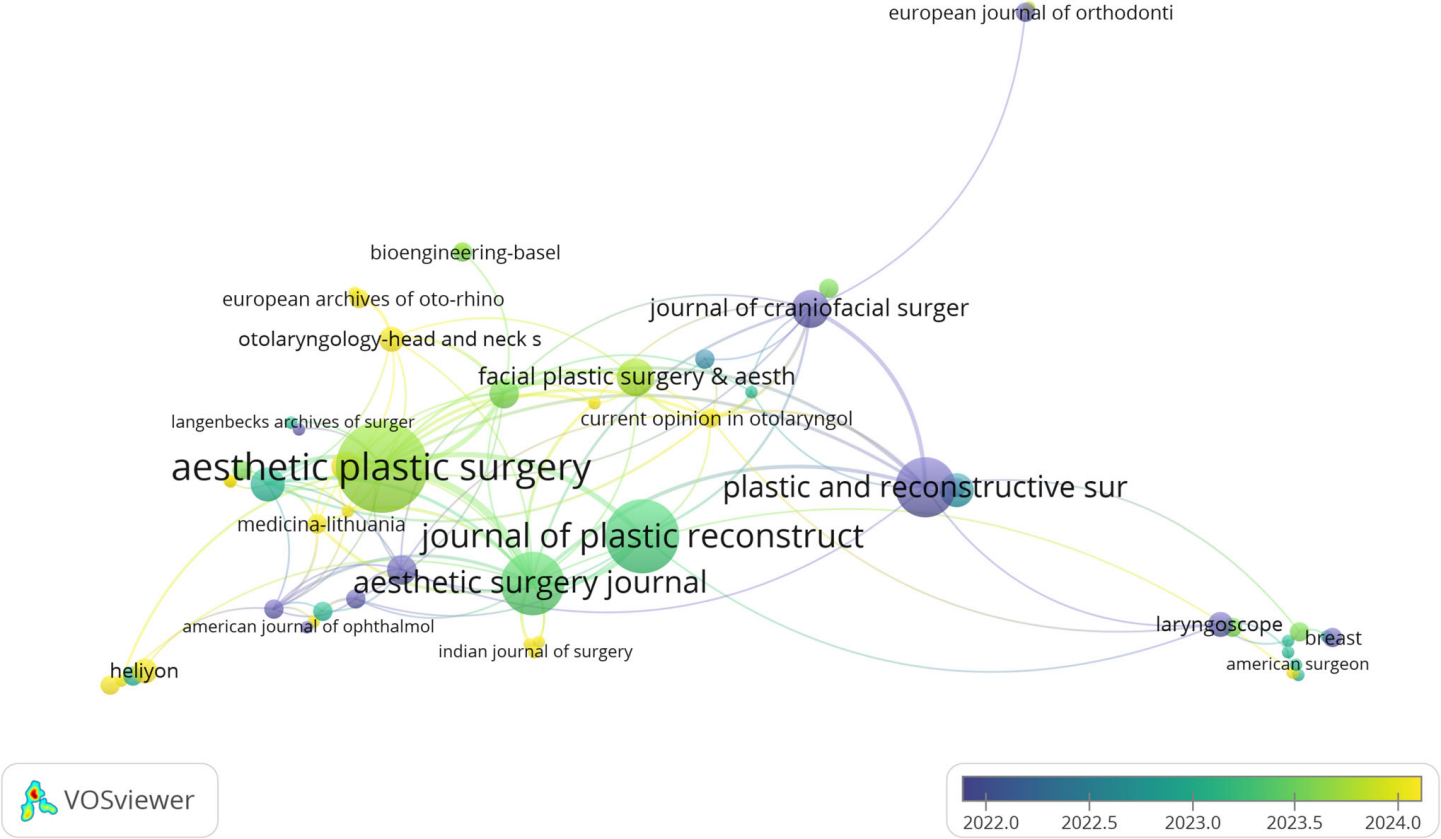
Analysis diagram of journal co-citation network. The size of the nodes indicates the number of published articles. The larger the node is, the more literature will be published

Using VOSviewer, we analyzed the publication volume and academic influence of the journals to identify the core journals in this field (Fig. 6 and Table 4). Although *Advanced Materials* only published one related article, it ranked first in terms of citation frequency, highlighting its huge influence. *Plastic and Reconstructive Surgery*, a long-standing authority, was the earliest to publish seminal work in this domain. Traditional plastic surgery journals such as *Aesthetic Plastic Surgery* and *Aesthetic Surgery Journal* also featured prominently.

**Table 4:** Journals with the top 10 citation numbers

Journal	Citations	Documents
Advanced Materials	288	1
Aesthetic Plastic Surgery	221	26
Aesthetic Surgery Journal	280	14
American Journal of Ophthalmology	19	1
American Journal of Otolaryngology	10	1
American Journal of Sports medicine	40	1
American surgeon	17	1
Annals of Biomedical Engineering	3	1
Annals of Plastic Surgery	43	5
Annals of Surgery	12	1

Beyond the key contributors profiled in the preceding tables, this investigation has identified and compiled additional forces advancing the field; the corresponding raw data reside in the supplementary materials.

### Keywords Cluster and Keywords Analysis

#### Keywords Cluster Analysis

**Fig. 7:**
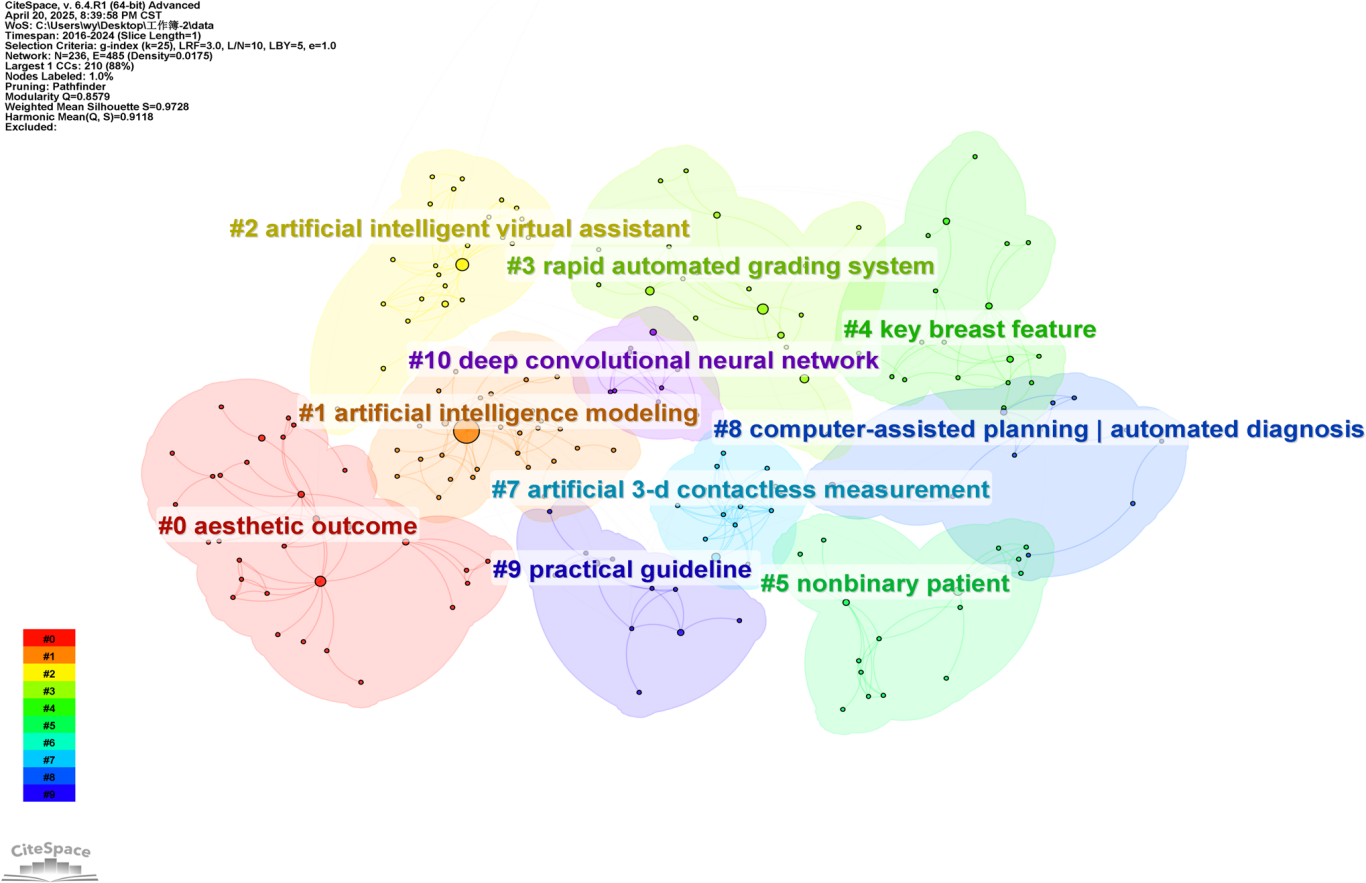
Analysis of keywords clustering. It was conducted by selecting the “cluster” option and employing the Pathfinder algorithm to draw connection lines and ensure the rationality of cluster classification. A total of 12 keywords clusters were identified, with each cluster assigned a distinct color based on the timestamp in the bottom left corner. The names of the clusters were derived from a set of representative keywords obtained using the LLR algorithm

In this study, CiteSpace was utilized to group keywords by selecting the "cluster". The results are illustrated in Fig. 7 in detail, which depicts the research topics related to the field with a total of 12 clusters.

These clusters are as follows: #0 aesthetic outcome (2021), #1 artificial intelligence modeling (2022), #2 artificial intelligent virtual assistant (2022), #3 rapid automated grading system (2021), #4 key breast feature (2022), #5 nonbinary patient (2022), #6 facial plastic (2019), #7 artificial 3-d contactless measurement (2016), #8 computer-assisted planning | automated diagnosis (2020), #9 practical guideline (2024), #10 deep convolutional neural network (2020), #12 workload (2023).

#### Keywords Analysis

In this study, we applied the burst detection algorithm in CiteSpace to visualize the evolution of research hotspots related to artificial intelligence (AI) applications in plastic and aesthetic surgery within the Web of Science database.

**Fig. 8:**
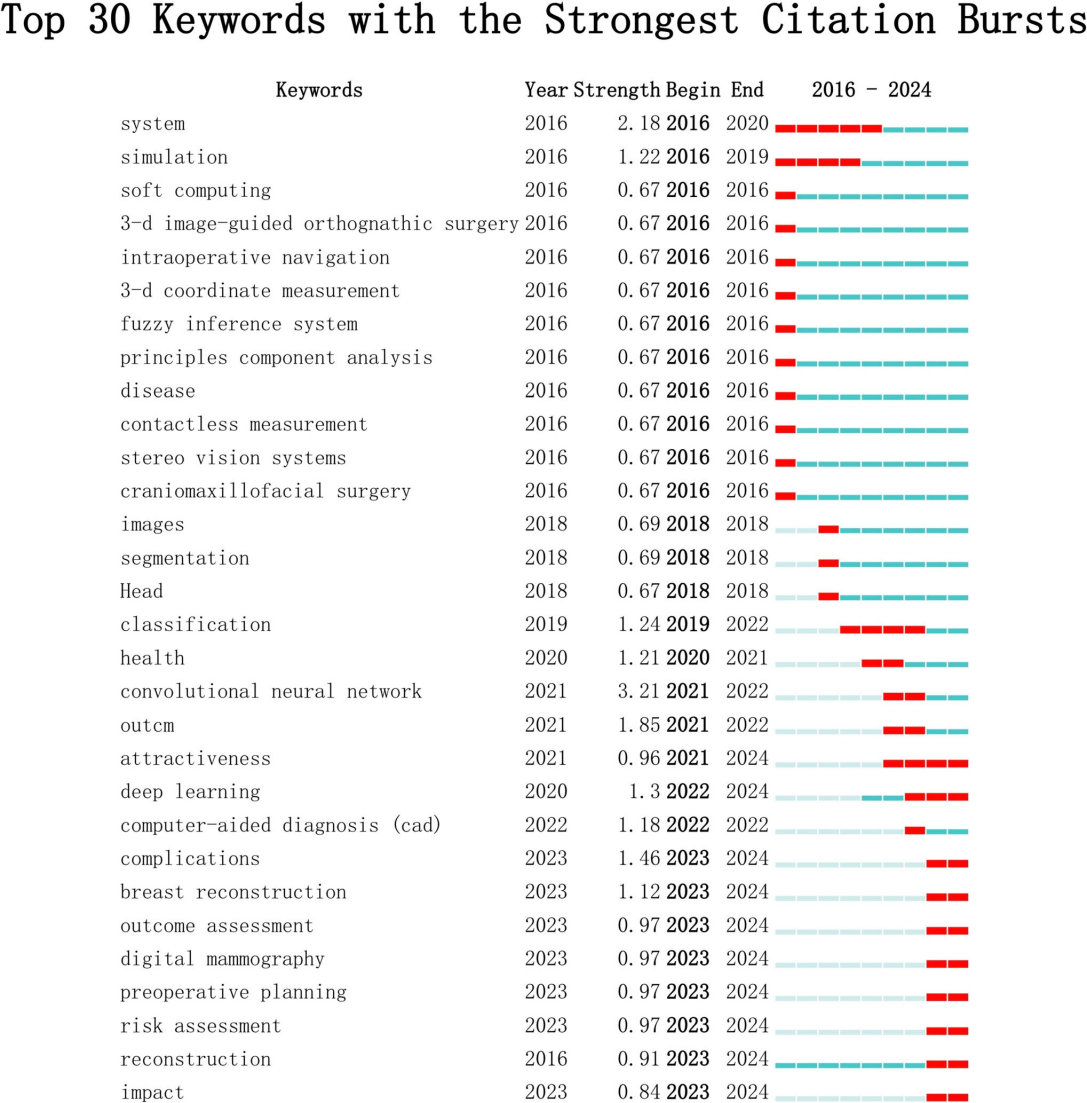
Top 30 keywords with the strongest citation bursts. The blue line represents the time span, while embedded red line within indicates the duration of the keyword burst

The generated map presents the top 30 keywords, illustrating their burst intensity and duration, as depicted in Fig. 8. At present, the most significant keywords include “attractiveness”, “deep learning”, “complications”, “breast reconstruction”, “outcome assessment”, “digital mammography”, “preoperative planning”, “risk assessment”, “reconstruction”, and “impact”.

A comprehensive analysis of keywords and keywords cluster was also performed. The detailed findings are presented in the supplementary materials.

## Discussion and Conclusion

This bibliometric study aims to summarize the research trends and hotspots in AI applications for plastic surgery while interpreting their societal significance.

Based on our analysis of the results, we have identified the countries, institutions, and scholars making outstanding contributions within this domain. The term "artificial intelligence" was first proposed at the 1956 Dartmouth Conference in the USA. Core conference scientists represented institutions such as Stanford and Harvard [33, 34]. The USA—home to tech giants including Silicon Valley companies and Microsoft—has maintained AI leadership through policy support and substantial funding [35]. China has rapidly advanced AI research in recent years, leveraging its population scale to generate robust datasets for training plastic surgery AI models. However, limited international collaboration may constrain the depth, breadth, and innovation of Chinese research. Japan's contributions to parallel computing and knowledge engineering stem from its 1980s–1990s Fifth Generation Computer Systems Project (FGCS), continuing to influence its role in AI-driven plastic surgery [9, 36]. Generally, institutional and author distribution reflects national trends, but the limited collaborative networks suggest the field remains nascent. Pioneering ideas concentrate among elite scientists and institutions—a pattern driven by talent shortages and technical barriers that currently hinder broader dissemination.

Nevertheless, middle- and low-income countries hold particular significance in advancing equitable access to AI technologies within cosmetic surgery, extending beyond traditionally research-intensive regions. Undeniably, nations without published contributions fall outside this analysis. While indicative of underdeveloped research systems in these regions, this omission impedes comprehensive assessment of geographic disparities' field impact. Crucially, economic constraints exacerbate regional inequities, introducing sampling bias in AI training data. Plastic surgery AI advancement requires engagement from low- and middle-income countries (LMICs). Tools trained on homogeneous datasets (e.g., Caucasian facial norms) risk misdiagnosing or excluding underserved populations.

To counteract systemic inequities, future priorities should lower technical barriers to accelerate AI adoption in developing nations and establish international data consortia enabling open-access, cross-ethnic algorithm validation.

Historically, Caucasian physical and facial characteristics have been consistently regarded as the aesthetic ideal [37]. Contemporary evidence demonstrates, however, that attractiveness perceptions and beauty standards exhibit significant variations across cultural backgrounds, ethnic groups, and geographical regions. Consequently, AI paradigms trained exclusively on Western big data lack universal applicability. Illustrative examples include Chinese facial aesthetics favoring soft contours and balanced features over angular definitions and full lips predominant in Western standards. Black and Hispanic women demonstrate stronger preferences for increased gluteal volume compared to White women, with European, Asian, and North American populations following this order of preference [37]. In breast augmentation procedures, Asian women typically seek non-ptotic, proportionally appropriate breast morphology—distinct from Western aesthetic norms [38]. Critically, surgical planning that incorporates culturally and ethnically specific aesthetic parameters is more likely to achieve optimal postoperative outcomes [37, 39].

Crucially, AI model training demonstrates significant dependency on input data quality. Medical imaging research on pneumonia diagnosis revealed a 34% underrepresentation of non-Caucasian samples in training data, directly contributing to a 22% reduction in F1-score for pneumonia detection in dark-skinned patients (Fitzpatrick skin types IV-VI). This statistically significant performance disparity confirms that skewed data distributions induce spurious correlations in algorithmic learning [40]. Further evidence emerges from a deep learning study utilizing chest radiographs: while achieving an area under the receiver operating characteristic curve (AUC-ROC) of 0.84 on Stanford Medical Center data, the identical model's performance declined to 0.69 when validated against community hospital datasets [41], demonstrating that data quality fundamentally supersedes quantity. Real-world clinical deployment introduces substantial uncontrolled variables—including phenotypic heterogeneity, operator-dependent variability, and medical imaging equipment tier discrepancies—which propagate artifactual associations in synthetic data. These constraints present formidable implementation barriers, particularly in resource-limited settings. Ultimately, the representativeness and integrity of training datasets critically determine the validation standards for aesthetic surgery AI models. We contend that cosmetic interventions should preserve individualized natural feature harmony. Therefore, clinically valid aesthetic benchmarks must derive from ethnogeographically diverse data repositories, necessitating expanded multicenter research consortia and deepened cross-disciplinary collaboration.

Moreover, our analysis identifies a critical concern within this domain: markedly deficient collaborative engagement across research entities, spanning geographical and institutional boundaries. Firstly, the interdisciplinary integration of AI and plastic surgery necessitates specialized expertise, further compounding collaborative challenges. Current impediments to international cooperation originate from structural complexities: institutional research outputs possess significant commercialization potential, inevitably leading to monopolization of core technologies and data assets, while intensifying academic competition has prompted stricter governmental data sovereignty regulations globally. Concurrently, structural barriers in cross-disciplinary funding perpetuate siloed grant allocation mechanisms, resulting in frequent rejection of interdisciplinary projects due to ambiguous domain delineation. Nevertheless, bibliometric evidence indicates that cultivating international and transdisciplinary partnerships remains critical for future advancements. Resolving this predicament requires synergistic efforts to establish constructive competitive frameworks and sustainable research ecosystems. We propose revising institutional and scholarly evaluation systems to incorporate quantifiable collaboration metrics. Academic journals should proactively create dedicated interdisciplinary sections, prioritizing publication of clinically validated AI-medical research jointly authored by surgical-engineering teams.

National research funding platforms should expand eligibility criteria for interdisciplinary collaborative projects and co-develop international data-sharing infrastructures. Neutral international bodies—including the World Health Organization (WHO), United Nations Educational, Scientific and Cultural Organization (UNESCO), and Association for Computing Machinery (ACM)—must execute their institutional mandates to facilitate cross-border, multidisciplinary knowledge integration. This requires implementing robust data security protocols and ethical governance frameworks to enable collaborative advancement of transparent artificial intelligence systems.

Through keyword co-occurrence analysis and emerging trend mapping, we have delineated the structural framework for AI integration in plastic and aesthetic surgery [42–44]. Artificial intelligence is projected to permeate the entire clinical workflow of cosmetic surgery—from preoperative diagnostic assessment to long-term prognostic monitoring—with AI-assisted decision systems demonstrating significant potential. Nevertheless, contemporary algorithms face multidimensional challenges.

While current AI performs adequately in common disease diagnosis, substantial improvement is required for scenarios involving insufficient data inputs, inadequate training, and image recognition challenges. Large language models (LLMs) and generative AI systems (e.g., ChatGPT and DeepSeek) exhibit capabilities to generate personalized diagnostic-therapeutic pathways through multimodal data integration (electronic health records, imaging reports, genomics). For instance, ChatGPT achieves 87.5% diagnostic accuracy for common dermatological conditions, while automated patient education enhances treatment adherence by 35%. However, its misdiagnosis rate for Fitzpatrick skin type V-VI is 2.3 times higher than for type I-II, with rare disease recognition declining precipitously to 41.3% [45]. In ophthalmic applications, integrating intraocular imaging with genomic data elevates diagnostic specificity to 91.3%, yet fails to detect 19.7% of mild cyclitis cases. Critically, 67% of models disregard tuberculosis reactivation risks associated with JAK inhibitors [46]. Therefore, future initiatives should prioritize developing medical fact-verification engines and domain-adaptive fine-tuning techniques to mitigate AI hallucinations, thereby accelerating clinical implementation of healthcare AI.

Although researchers increasingly address personalized needs (e.g., gender-diverse populations), the persistent prominence of the burst keyword "attractiveness" raises a critical question: Can AI truly adapt aesthetic standards across different population [47, 48]? This concern highlights previously discussed institutional and geopolitical factors, particularly the dominance of Western countries in computational resources and biomedical data resource. Such technological concentration has hindered equitable progress, while the reliance on predominantly Western clinical cohorts introduces regional biases into AI training datasets. As a result, current AI-driven aesthetic models often lack transnational applicability [49, 50].

Furthermore, clusters like "rapid automated grading systems" and burst terms including "preoperative planning" indicate dual outcomes: operational efficiency gains versus concerning overreliance on algorithmic guidance [48, 51, 52]. This dependency potentially erodes surgical autonomy during complex interventions where clinical judgment proves paramount. The core limitation remains AI's deficient decision-making depth compared to experienced clinicians [53]. Current systems frequently fail to adequately incorporate patient-specific variables or surgical subtleties and may generate hallucinated content [19, 55, 56].

Surgical contexts are extreme complexity—robotic systems rely on sensor-derived environmental interpretation, where suboptimal data acquisition and limited haptic feedback impair real-time adaptive decision-making. For example, during recurrent laryngeal nerve dissection in thyroidectomy, involuntary patient swallowing can lead to anatomical misinterpretation, risking irreversible neural injury [54]. Similarly, analyses of robotic hepatectomy have shown that vascular anomalies may cause AI navigation systems to misidentify and misligate hepatic arteries, with intraoperative hallucination presenting serious safety concerns.

Moreover, computational approaches often struggle to reconcile theoretical optima with physiological and contextual constraints inherent in real-world clinical scenarios, which are marked by complexity and sometimes conflicting parameters. While numerous studies have shown diagnostic AI performance comparable to human experts, surgical decision-making integrates both medical evidence and patients' socio-contextual backgrounds—an aspect that algorithmic systems are fundamentally limited in addressing. This limitations explains the currently restricted real-world integration of AI in clinical decision-making pathways [57, 58]

Besides, persistent challenges also include unresolved issues surrounding data privacy, algorithmic bias, and equitable deployment of AI-driven clinical tools. Importantly, AI's developmental trajectory and nascent capacity for ethical reasoning merit continued investigation. Addressing these gaps requires augmenting training datasets with globally representative data and implementing rigorous multidisciplinary validation to ensure clinical accuracy and relevance [59]. Ongoing concerns regarding data security and algorithmic transparency in AI-generated recommendations necessitate sustained attention. Future innovation must balance technological advancement with ethical imperatives [60]. Establishing a transparent, inclusive, and auditable AI healthcare ecosystem demands coordinated collaboration among clinicians, AI engineers, and ethicists—advancing precision surgery anchored in patient safety and demographic inclusivity.

While AI-driven aesthetic assessment systems demonstrate transformative potential in bridging objective metrics with subjective perceptions, our analysis cautions against premature declarations of clinical paradigm shifts. Current implementations remain in a nascent adoption phase, constrained by limited cross-cultural adaptability and insufficient real-world validation [61]. Transitioning from conceptual promise to practical implementation requires sustained refinement; surgeons and algorithm developers must prioritize patient-centered healthcare delivery while ensuring equitable resource allocation to maximize societal benefit from technological progress [62]. Strategic advancements should prioritize culturally representative datasets, develop explainable and auditable algorithmic architectures, establish multidisciplinary clinical validation frameworks, and leverage emerging technology synergies [63, 64]. Artificial intelligence should enhance rather than replace surgeons. This paradigm requires clinicians to strengthen critical thinking and problem-solving skills in AI-augmented workflows, elevate professional standards, and drive systemic improvements in efficiency and quality [65].

Although this is the first bibliometric analysis focusing on the application of AI in plastic surgery, there are still some limitations in this study, as described below:

1. We only conducted literature search in WOSCC. Although WOSCC is regarded as a reliable database for bibliometric analysis, some relevant literature may still be overlooked. However, using other databases may lead to issues such as incomplete citation records, low-quality academic outputs, incompatible information formats, and missing information. Additionally, WOSCC covers most of the high-quality literature in the medical field, which is why we chose it as the source database for this study.
2. We only examined English-language literature relevant to the field, which may introduce language bias. However, non-English literature will face challenges related to the information gap during the software analysis phase, and non-English publications account for less than 2% in the WOSCC database. Therefore, it is reasonable to exclude non-English literature.
3. Our literature search uses the topic search (TS) mode instead of the full-text search, which may result in the omission of some relevant literature. However, a full-text search may yield unrelated articles outside the scope of this field, significantly increasing the burden of unnecessary manual screening. In the future, integrating mature AI technologies for screening could enhance this process
4. The main purpose and significance of bibliometric analysis is to summarize the research hotspots and trends in the field, rather than to elucidate specific research findings.

## Supplementary Information

Below is the link to the electronic supplementary material.

Supplementary file 1 (XLSX 74 kb)

## Data Availability

Not applicable.

## Declarations

### Informed Publication

For this type of study informed consent in not required.

## References

[1] Asimakopoulou E, Zavrides H, Askitis T. The correlation of aesthetic plastic surgery with sexual, social, and romantic life in Cyprus. Plast Surg Nurs: Off J Am Soc Plast Reconstr Surg Nurs. 2020;40(2):100–5. 10.1097/PSN.0000000000000303 32459758

[2] Asimakopoulou E, Zavrides H, Askitis T. Plastic surgery on body image, body satisfaction and self-esteem. Acta Chir Plast. 2020;61(1–4):3–9. 32380836

[3] Perdikis G, et al. Aesthetic surgery in plastic surgery academia. Aesthetic Surg J. 2021;41(7):829–41. 10.1093/asj/sjaa181 32794545

[4] Tran DK, et al. Exploring the potential of stem cell-based therapy for aesthetic and plastic surgery. IEEE Rev Biomed Eng. 2023;16:386–402. 34905495 10.1109/RBME.2021.3134994

[5] Nyberg EL, et al. 3D-printing technologies for craniofacial rehabilitation, reconstruction, and regeneration. Ann Biomed Eng. 2017;45(1):45–57. 27295184 10.1007/s10439-016-1668-5 PMC5154778

[6] Moayer R, et al. Minimally invasive aesthetic procedures: an update on a decade-old prediction. Facial Plast Surg. 2017;33(4):434–6. 28753719 10.1055/s-0037-1603948

[7] Rahman E, et al. Sculpting digital identities: the interplay of aesthetic medicine, plastic surgery, and the metaverse. Eur J Plast Surg. 2023;46(6):845–54.

[8] Koljonen V. What could we make of AI in plastic surgery education. J Plast Reconstr Aesthet Surg. 2023;81:94–6. 37137194 10.1016/j.bjps.2023.04.055

[9] Kapetanios E. Quo Vadis computer science: from turing to personal computer, personal content and collective intelligence. Data Knowl Eng. 2008;67(2):286–92.

[10] Smith DM, et al. Applications of virtual reality in aesthetic surgery. Plast Reconstr Surg. 2005;116(3):898–904. 16141835 10.1097/01.prs.0000176901.37684.8a

[11] Korczak K, et al. A computer-supported management of photographic documentation in plastic surgery—System development and its clinical application. Comput Biol Med. 2017;86:1–5. 28494382 10.1016/j.compbiomed.2017.05.002

[12] Rahman E, et al. Skin, scalpel and the silicon chip: a systematic review on the accuracy, bias and data governance of artificial intelligence in dermatology, minimally invasive aesthetics, aesthetic, plastic and reconstructive surgery. Eur J Plast Surg. 2025;48(1):1.

[13] Nogueira R, et al. Machine learning, deep learning, artificial intelligence and aesthetic plastic surgery: a qualitative systematic review. Aesthetic Plast Surg. 2025;49(1):389–99. 39384606 10.1007/s00266-024-04421-3

[14] Gore JC. Artificial intelligence in medical imaging. Magn Reson Imaging. 2020;68:A1–A4. 31857130 10.1016/j.mri.2019.12.006

[15] Moglia A, et al. A systematic review on artificial intelligence in robot-assisted surgery. Int J Surg. 2021;95:106151. 34695601 10.1016/j.ijsu.2021.106151

[16] Yeh CC, et al. Implementing AI models for prognostic predictions in high-risk burn patients. Diagnostics (Basel). 2023;13(18). 10.3390/diagnostics13182984 PMC10528558 37761351

[17] Kiwan O, et al. Artificial intelligence in plastic surgery, where do we stand? Jpras Open. 2024;42:234–43. 39435018 10.1016/j.jpra.2024.09.003 PMC11491964

[18] Gorgy A, et al. Integrating AI into breast reconstruction surgery: exploring opportunities, applications, and challenges. Plast Surg (Oakv). 2024;22925503241292349. 10.1177/22925503241292349 PMC11559540 39545210

[19] Xie Y, et al. Evaluation of the artificial intelligence chatbot on breast reconstruction and its efficacy in surgical research: a case study. Aesthetic Plast Surg. 2023;47(6):2360–9. 37314466 10.1007/s00266-023-03443-7 PMC10784397

[20] Pacifico MD, et al. Preoperative planning for DIEP breast reconstruction: early experience of the use of computerised tomography angiography with VoNavix 3D software for perforator navigation. J Plast Reconstr Aesthet Surg. 2009;62(11):1464–9. 18708309 10.1016/j.bjps.2008.04.056

[21] Barbon C, et al. Exploring the learning curve of a new robotic microsurgical system for microsurgery. JPRAS Open. 2022;34:126–33. 36304073 10.1016/j.jpra.2022.09.002 PMC9593278

[22] Weinzierl A, et al. Benefits of robotic-assisted lymphatic microsurgery in deep anatomical planes. JPRAS Open. 2023;37:145–54. 37546233 10.1016/j.jpra.2023.07.001 PMC10403710

[23] Borsting E, et al. Applied deep learning in plastic surgery: classifying rhinoplasty with a mobile app. J Craniofac Surg. 2020;31(1):102–6. 31633665 10.1097/SCS.0000000000005905

[24] Webb WR, et al. Harmony and hype: navigating translational science in aesthetic medicine and plastic surgery. Eur J Plast Surg. 2024;47(1).

[25] Zhang F, et al. Bibliometric and visual analysis of fecal microbiota transplantation research from 2012 to 2021. Front Cell Infect Microbiol. 2022;12:1057492. 36439220 10.3389/fcimb.2022.1057492 PMC9684174

[26] Zhang J, et al. Bibliometric and visualized analysis of arthroscopic treatment of acromioclavicular joint injury. J Orthop Surg Res. 2023;18(1):728. 37752567 10.1186/s13018-023-04193-7 PMC10523771

[27] Zhang L, et al. Worldwide research trends on tumor burden and immunotherapy: a bibliometric analysis. Int J Surg. 2024;110(3):1699–710. 38181123 10.1097/JS9.0000000000001022 PMC10942200

[28] Ellegaard O, Wallin JA. The bibliometric analysis of scholarly production: How great is the impact? Scientometrics. 2015;105(3):1809–31. 26594073 10.1007/s11192-015-1645-z PMC4643120

[29] Pan XL, et al. Examining the usage, citation, and diffusion patterns of bibliometric mapping software: a comparative study of three tools. J Informet. 2018;12(2):481–93.

[30] Chen CM. CiteSpace II: detecting and visualizing emerging trends and transient patterns in scientific literature. J Am Soc Inform Sci Technol. 2006;57(3):359–77.

[31] Wang LY, et al. Way to accomplish low carbon development transformation: a bibliometric analysis during 1995–2014. Renew Sustain Energy Rev. 2017;68:57–69.

[32] Triana L, et al. Trends in surgical and nonsurgical aesthetic procedures: a 14-year analysis of the international society of aesthetic plastic surgery-ISAPS. Aesthetic Plast Surg. 2024;48(20):4217–27. 39103642 10.1007/s00266-024-04260-2

[33] Moor J. The Dartmouth College artificial intelligence conference: the next fifty years. AI Mag. 2006;27(4):87–91.

[34] Cordeschi R. AI turns fifty: revisiting its origins. Appl Artif Intell. 2007;21(4–5):259–79.

[35] Madhavan R, et al. Toward trustworthy and responsible artificial intelligence policy development. IEEE Intell Syst. 2020;35(5):103–8.

[36] Garvey C. Artificial intelligence and Japan’s fifth generation: the information society, neoliberalism, and alternative modernities. Pac Hist Rev. 2019;88(4):619–58.

[37] Arian H, et al. Cosmetic surgery and the diversity of cultural and ethnic perceptions of facial, breast, and gluteal aesthetics in women: a comprehensive review. Clin Cosmet Invest Dermatol. 2023;16:1443–56. 10.2147/CCID.S410621 PMC10258039 37313510

[38] Swami V, et al. The attractive female body weight and female body dissatisfaction in 26 countries across 10 World Regions: Results of the International Body Project I. Pers Soc Psychol Bull. 2010;36(3):309–25. 20179313 10.1177/0146167209359702

[39] Perrett DI, May KA, Yoshikawa S. Facial shape and judgements of female attractiveness. Nature. 1994;368(6468):239–42. 8145822 10.1038/368239a0

[40] Kambhatla G, Stewart I, Mihalcea R. Surfacing racial stereotypes through identity portrayal. In: Proceedings of the 2022 ACM Conference on Fairness, Accountability, and Transparency. 2022, Association for Computing Machinery: Seoul, Republic of Korea. p. 1604–1615.

[41] Zech JR, et al. Variable generalization performance of a deep learning model to detect pneumonia in chest radiographs: a cross-sectional study. PLoS Med. 2018;15(11): e1002683. 30399157 10.1371/journal.pmed.1002683 PMC6219764

[42] Ratten V. Artificial intelligence, digital trends and globalization: future research trends. FIIB Bus Rev. 2024;13(3):286–93.

[43] Chopra H, et al. Artificial intelligence in surgery: modern trends. Int J Surg. 2022;106. 10.1016/j.ijsu.2022.106883 PMC9444844 36075553

[44] Prado AS. The fourth industrial revolution tackles plastic surgeons. Plast Reconstr Surg. 2018;142(5):821E-822E. 30119128 10.1097/PRS.0000000000004914

[45] Lewandowski M, et al. A systemic review of large language models and their implications in dermatology. Austral J Dermatol. 2025;66(4):e202–8. 10.1111/ajd.14484 40189745

[46] Tan Yip Ming C, et al. The potential role of large language models in uveitis care: perspectives after ChatGPT and Bard Launch. Ocul Immunol Inflamm. 2024;32(7):1435–9. 37562028 10.1080/09273948.2023.2242462

[47] Ozmen BB, Schwarz GS. Future of artificial intelligence in plastic surgery: toward the development of specialty-specific large language models. J Plast Reconstr Aesthet Surg. 2024;93:70–1. 38670034 10.1016/j.bjps.2024.04.054

[48] Park KW, et al. Artificial intelligence in facial plastics and reconstructive surgery. Otolaryngol Clin North Am. 2024;57(5):843–52. 38971626 10.1016/j.otc.2024.05.002

[49] Musih N, Fisher E. Dialectics of training: A critique of recommendation engines' aesthetic judgment. Converg-Int J Res New Media Technol. 2024.

[50] Mondol T, Brown DG. Computational creativity and aesthetics with algorithmic information theory. Entropy. 2021;23(12). 10.3390/e23121654 PMC8700213 34945960

[51] Vles MD, et al. Virtual and augmented reality for preoperative planning in plastic surgical procedures: a systematic review. J Plast Reconstr Aesthet Surg. 2020;73(11):1951–9. 32622713 10.1016/j.bjps.2020.05.081

[52] Jin MY, et al. Three-dimensional scanning for breast plastic and reconstructive surgery: an updated review. Eur J Plast Surg. 2024;47(1).

[53] Buzzaccarini G, Degliuomini RS, Borin M. The artificial intelligence application in aesthetic medicine: how ChatGPT can revolutionize the Aesthetic World. Aesthetic Plast Surg. 2023;47(5):2211–2. 37256297 10.1007/s00266-023-03416-w

[54] Rivero-Moreno Y, et al. Autonomous robotic surgery: Has the future arrived? Cureus J Med Sci. 2024;16(1). 10.7759/cureus.52243 PMC10862530 38352080

[55] Farid Y, et al. Artificial intelligence in plastic surgery: insights from plastic surgeons, education integration, ChatGPT's survey predictions, and the path forward. Plast Reconstr Surg-Glob Open. 2024;12(1). 10.1097/GOX.0000000000005515 PMC10781127 38204870

[56] Citron I, et al. Fact or fake news: What are AI chatbots telling our patients about aesthetic surgery? J Plast Reconstr Aesthet Surg. 2023;86:280–7. 37797376 10.1016/j.bjps.2023.09.033

[57] Holm S. Handle with care: assessing performance measures of medical AI for shared clinical decision-making. Bioethics. 2022;36(2):178–86. 34427331 10.1111/bioe.12930

[58] Rad AA, et al. The ethical considerations of integrating artificial intelligence into surgery: a review. Interdiscip Cardiovasc Thorac Surg. 2025;40(3). 10.1093/icvts/ivae192 PMC11904299 39999009

[59] Choi E, et al. Artificial intelligence in facial plastic surgery: a review of current applications, future applications, and ethical considerations. Facial Plast Surg. 2023;39(05):454–9. 37353051 10.1055/s-0043-1770160

[60] Kenig N, Echeverria JM, Rubi C. Ethics for AI in plastic surgery: guidelines and review. Aesthetic Plast Surg. 2024;48(11):2204–9. 38456892 10.1007/s00266-024-03932-3

[61] Abi-Rafeh J, et al. Preoperative patient guidance and education in aesthetic breast plastic surgery: a novel proposed application of artificial intelligence large language models. Aesthetic Surg J Open Forum 2024;6. 10.1093/asjof/ojae062 PMC11385898 39257998

[62] Chappell AG, Teven CM. How should surgeons consider emerging innovations in artificial intelligence and robotics? AMA J Ethics. 2023;25(8):E589-597. 37535503 10.1001/amajethics.2023.589

[63] Thirunavukarasu AJ, et al. Large language models in medicine. Nat Med. 2023;29(8):1930–40. 37460753 10.1038/s41591-023-02448-8

[64] Weiss M, Tonella P. Adopting two supervisors for efficient use of large-scale remote deep neural networks - RCR Report. ACM Trans Softw Eng Methodol. 2023;33(1):Article 29.

[65] Lim B, et al. Exploring the unknown: evaluating ChatGPT’s performance in uncovering novel aspects of plastic surgery and identifying areas for future innovation. Aesthetic Plast Surg. 2024;48(13):2580–9. 38528129 10.1007/s00266-024-03952-z PMC11239602

